# Clinical and diagnostic imaging outcomes of mandibular fracture management in 109 cats

**DOI:** 10.3389/fvets.2025.1633636

**Published:** 2025-07-23

**Authors:** Janny V. Evenhuis, Anna Vincek, Stephanie Goldschmidt, Maria Soltero-Rivera, Mindy A. Nguyen, Boaz Arzi

**Affiliations:** ^1^School of Veterinary Medicine, William R. Pritchard Veterinary Medical Teaching Hospital, University of California, Davis, Davis, CA, United States; ^2^Department of Surgical and Radiological Sciences, University of California, Davis, Davis, CA, United States

**Keywords:** computed tomography, cone beam CT, fracture, bone healing, temporomandibular joint, skull, facial trauma, feline

## Abstract

**Introduction:**

Mandibular injuries are a common occurrence in cats that are presented for maxillofacial trauma. Timely assessment and treatment of these injuries directly impacts a cat’s return to function.

**Methods:**

A retrospective study was performed on a population of 109 cats that were presented for evaluation and treatment of mandibular trauma. Medical records and diagnostic imaging were reviewed to determine mandibular fracture location, morphology, and treatment. Follow-up data were obtained from repeat clinical examination and diagnostic imaging.

**Results:**

The most commonly injured anatomical locations were the mandibular symphysis (55.0%), the condylar process of the mandible (49.5%) and mid ramus (48.6%). More severe pre-operative fracture displacement was associated with a poor healing outcome in the mid ramus and coronoid process regions. The group of cats treated with open reduction and internal fixation (ORIF) had a significantly higher percentage of cats showing adequate healing (*P* = 0.0247) compared to the group of cats treated with maxillomandibular fixation (MMF). Cats treated with ORIF also had lower prevalence of persistent malocclusion (9.1%) when compared to cats treated with MMF (53.9%) (*P* = 0.0138, respectively). Placement of an esophageal feeding tube did not have a statistically significant impact on weight change in patients post-operatively (*P* = 0.0973). Patient survival was high at 94.5%.

**Discussion:**

High patient survival indicates that cats that were diagnosed and treated for mandibular trauma often have a good prognosis. Pre-operative fracture displacement may influence healing in select regions of the mandible. Fractures treated with ORIF had a higher rate of adequate bone healing when compared with fractures treated with MMF.

## 1 Introduction

Maxillofacial trauma is a common and debilitating condition in cats and an estimated 14.5–34% of all traumatic injuries in cats affect the maxillofacial region (1, 2). The most common causes of maxillofacial injuries in cats include automobile-related trauma (39.5–89%), altercations with other animals (4.4–27.3%), fall from height (1–3%), and ballistic trauma (1%) (1–6). Possible outcomes of damage to the osseous structures of the maxillofacial region include defect non-union, malunion, infection, pain, persistent malocclusion, and loss of function of the masticatory apparatus. In addition to damage to the tooth bearing regions of the masticatory apparatus, temporomandibular joint (TMJ) injury is commonly observed in cats with maxillofacial injuries and may result in intra- or extra-articular ankylosis, septic arthritis, chronic arthritis, mandibular drift, limited TMJ range of motion and chronic facial pain (1, 7–10). Furthermore, feline maxillofacial trauma patients frequently have concurrent soft tissue injury including neurologic, ocular, pharyngeal, and tongue injury. Mortality due to maxillofacial trauma has varied greatly depending on the population studied (8–37.7%) and survival may ultimately depend on the extent of the injury, neurologic evaluation at the time of presentation, and access to veterinary care (6, 11, 12). Furthermore, quality of healing after maxillofacial trauma is an essential but understudied topic in domestic cats, as this directly impacts the quality of life and functional capacity of these patients.

Historically, fixation of midface fractures in cats has been challenging due to the small size and thin structure of the bones in this region, which makes it difficult to secure implants effectively. Therefore, much of the research discussing treatment of feline maxillofacial trauma has focused on fixation of injuries of the mandible (1, 3–5). Reported methods of fixation of mandibular injuries include circummandibular cerclage wire, rigid or elastic maxillo-mandibular fixation (MMF), interdental wire and composite splints, interfragmentary wire, arch bars, muzzle coaptation, external fixators, and open reduction and internal fixation (ORIF) (13–17). Despite the availability of various methods and approaches, the literature lacks robust and comparative patient follow-up with repeated advanced imaging using conventional computed tomography (CT) or cone beam computed tomography (CBCT) for evaluation of fracture healing. For this reason, it is difficult to ascertain the success or failure rates of any one method of fixation.

The primary goals of treatment of maxillofacial trauma are to restore the patient occlusion and return to normal comfort and function in the most efficient manner, while mitigating the need for repeat procedures and therapies (18). In this retrospective study, we hypothesized that mandibular fracture configuration and choice in method of fixation would correlate with quality of healing and return to normal function. Thus, our first aim was to characterize mandibular fracture patterns in cats presenting to a tertiary referral hospital for maxillofacial trauma. A second aim of the study was to document the method of fixation used for treatment of mandibular fractures and to assess whether treatments impacted fracture healing outcome.

## 2 Materials and methods

### 2.1 Case selection

The electronic medical record database of the University of California - Davis Veterinary Medical Teaching Hospital was searched for cats that were presented for maxillofacial trauma between the years 2013 to 2024. For inclusion, cats must have received advanced diagnostic imaging of the skull in the form of conventional CT or CBCT at the initial visit. Cats must have been diagnosed with at least one mandibular fracture or symphyseal separation. Cats must have sustained injuries within 7 days of presentation to be included in the study. There were no limitations on signalment parameters of cats included in the study. Exclusion criteria included incomplete medical records, CT with >0.5 mm slice thickness, and chronic maxillofacial injuries older than 7 days from the time of assessment. No specific follow-up imaging or recheck examinations were required for initial inclusion. However, only cats with at least 1 follow-up examination within 2–4 weeks were considered for outcome data such as complications due to fracture fixation or injury, residual pain, infection, dehiscence and persistence of malocclusion. Patient history from follow-up medical records as reported by the owner was reviewed for evidence of complications or diminished function related to the previous trauma or treatment. Furthermore, cats were only included for fracture healing data if they underwent repeat CBCT, CT, or radiographic assessment.

### 2.2 Image acquisition and evaluation

All cats underwent advanced imaging at their initial visit with CBCT (NewTom 5G CBCT Scanner, NewTom, Verona, Italy) and/or conventional CT (HiSpeed FX/i or LightSpeed16, GE Healthcare, Waukesha, WI). Conventional CT was mainly utilized for patients with moderate to marked soft tissue trauma especially of the pharyngeal region, tongue, or periocular region and for patients who had high suspicion of neurological or ocular rupture. DICOM files from each study were viewed using a DICOM viewer (Agfa HealthCare Enterprise Imaging, 580 Gotham Parkway, NJ 07072 Carlstadt, USA) and viewed dynamically on medical flat-grade monitors (ASUS PB278Q 27-inch, ASUSTeK Computer Inc., Taipei, Taiwan). This allowed the observers to utilize multiplanar reconstruction (MPR) and 3-dimensional (3D) reconstruction capabilities on all cases. A board-certified veterinary radiologist (AV) and board-certified veterinary dentist (JVE) collectively viewed all studies and recorded all the imaging data.

### 2.3 Fracture evaluation

**Figure 1:**
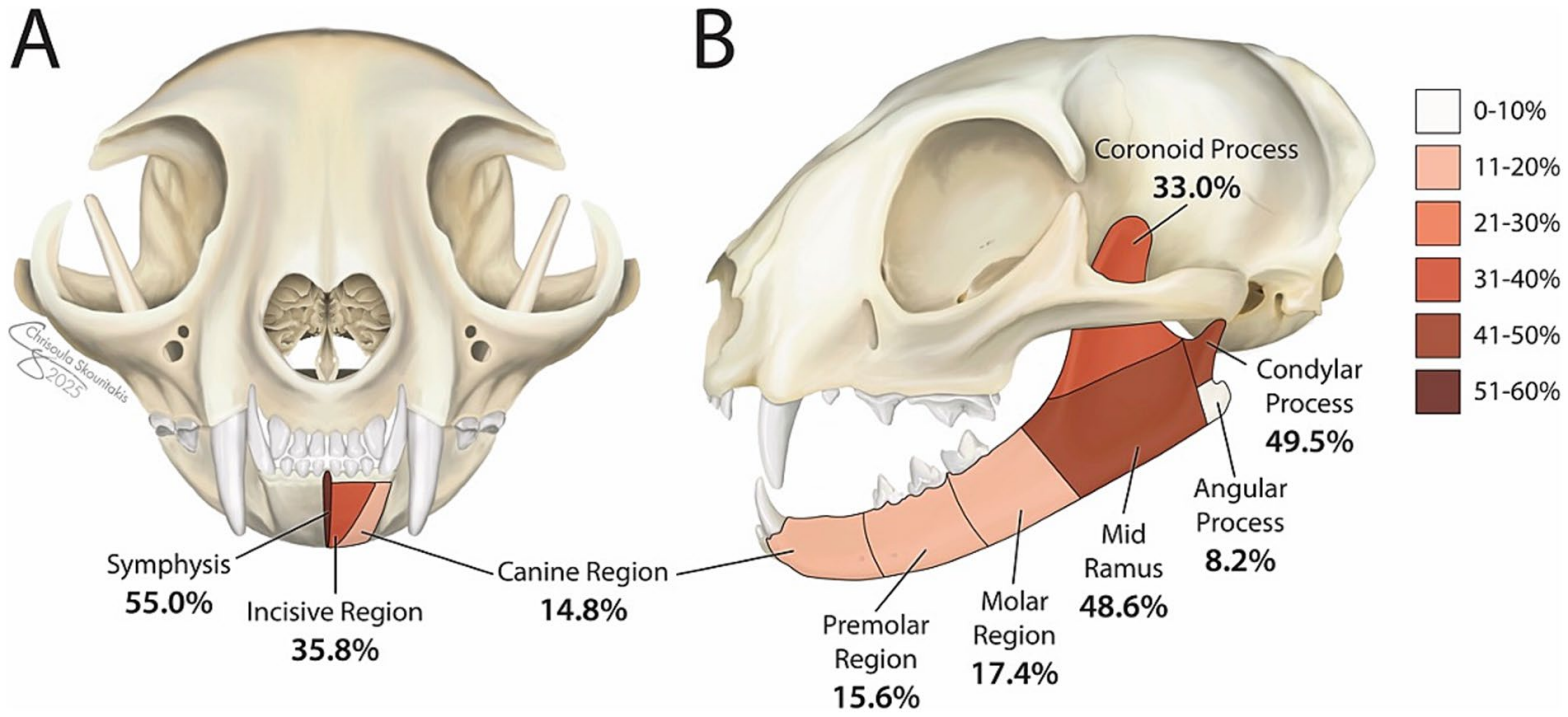
Summary of prevalence of injured regions of the mandible in the cohort of cats. Each percentage represents the percentage of cats that exhibited a fracture in each respective region affecting the left or right mandible or both mandibles. Coloration of each segment indicates frequency of injury with darker colors denoting more commonly injured regions. ‘Symphysis’ denotes the percentage of cats exhibiting symphyseal separation.

**Figure 2:**
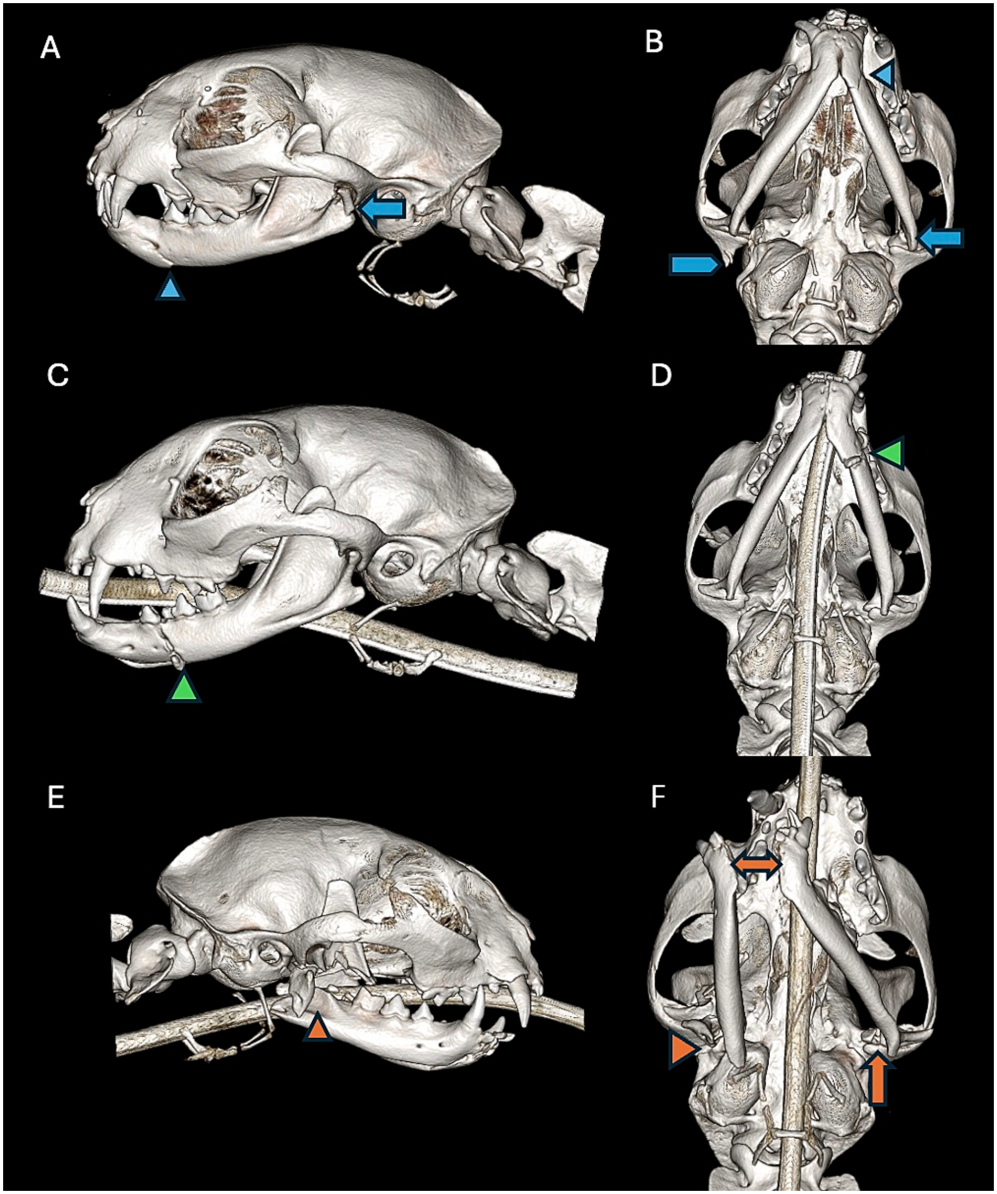
Left lateral view **(A)** and a ventral-dorsal view **(B)** of a CBCT 3D reconstruction image of a cat exhibiting a non-displaced fracture of the canine region of the left mandible (blue arrowhead). There is also a severely displaced fracture of the condylar process of the left mandible (blue arrow) and a severely displaced fracture of the right squamous temporal bone (blue pentagon). Left lateral view **(C)** and a ventral- dorsal view **(D)** of a CBCT 3D reconstruction of a cat exhibiting a mildly displaced fracture of the premolar region of the left mandible (green arrowhead). Right lateral view **(E)** and a ventral-dorsal view **(F)** of a CBCT 3D reconstruction of a cat exhibiting a severely displaced fracture involving the mid ramus, coronoid process, and angular process of the right mandible (orange arrowhead). The orange arrowhead depicts a severely displaced fracture involving the mid ramus, coronoid process, and angular process of the right mandible. The double headed orange arrow depicts severe displacement of the mandibular symphysis. There is also a concurrent mildly displaced fracture of the condylar process of the left mandible (orange arrow).

**Figure 3:**
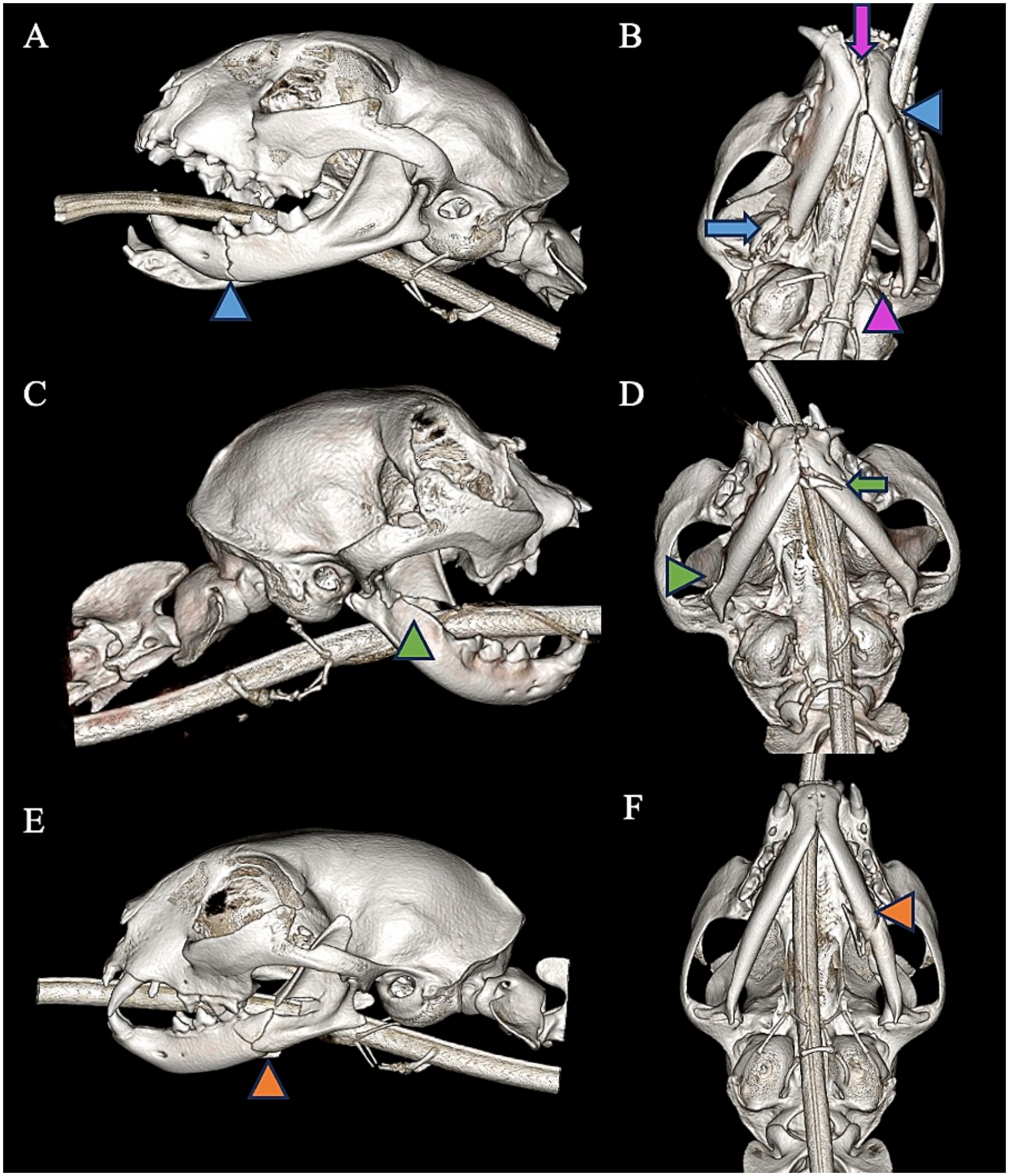
Left lateral view **(A)** and a ventral-dorsal view **(B)** of a CBCT 3D reconstruction of a cat with exhibiting an incomplete fracture of the premolar region of the left mandible (blue arrowhead). There are also concurrent fractures of mid ramus of the right mandible (blue arrow) and condylar process of the left mandible (purple arrowhead) as well as symphyseal separation (purple arrow). Right lateral view **(C)** and a ventral-lateral **(D)** of a CBCT 3D reconstruction of a cat exhibiting a complete, non-fragmented fracture of the coronoid process of the right mandible (green arrowhead). There is also a concurrent complete, fragmented fracture of the canine and premolar region of the left mandible (green arrow). Left lateral view **(E)** and ventral-lateral view **(F)** of a 3D reconstruction of a cat with a complete, fragmented fracture of the mid ramus and molar regions of the left mandible (orange arrowhead).

Each cat’s mandible was divided into specific bones and regions as previously described (19) (Figure 1). For each bone and region, it was determined whether each bone or region was fractured. If so, fracture morphology was described in terms of displacement and fragmentation. The degrees of displacement and fragmentation were modeled after the AOCMF fracture classification system in humans (Table 1) (20). For both displacement and fragmentation, a score of 0 indicated no fracture. When scoring displacement, a score of 1 indicated no displacement, a score of 2 minimal displacement with ≥50% overlap remaining between fragments, and a score of 3 severe displacement with <50% overlap remaining (Figures 2A–F). When scoring fragmentation, a score of 1 indicated an incomplete fracture, a score of 2 a complete fracture, and a score of 3 a comminuted fracture (Figures 3A–F). A comminuted fracture was defined as a fracture having 3 or more bone fragments.

**Table 1:** Fracture characterization scheme describing classification for displacement and fragmentation based on pre-operative CT or CBCT imaging.

Score	Displacement	Fragmentation
0	No fracture	No fracture
1	No displacement	Incomplete fracture
2	Mild displacement; ≥50% overlap remaining between fragments	Complete fracture without comminution
3	Severe displacement; <50% overlap remaining between fragments	Complete fracture with comminution

The fracture was determined to be unilateral or bilateral in nature. If the fracture was bilateral, the highest allocated ordinal value for fragmentation and displacement was used for statistical analysis. Symphyseal separation was considered separately from other traumatic injury.

If a fracture occurred at a border between two regions, the bone or region on both sides was considered fractured, and the morphology of the fracture was considered separately for each bone or region.

### 2.4 Fracture etiology and concomitant trauma

The cause of maxillofacial trauma was recorded if it was directly witnessed. The cause of trauma was marked as unknown if it was not witnessed. Concurrent maxillary trauma and/or dentoalveolar trauma as noted in oral exam or on diagnostic imaging and was recorded as present or absent. Concomitant trauma to the limbs, thoracic cavity, abdominal cavity, and skin were also recorded. Evidence of traumatic brain injury characterized by decrease in mentation, ataxia, anisocoria, and changes to blood pressure and heart rate indicating a Cushing’s reflex were also recorded. Presence of ocular trauma as characterized by corneal ulceration, scleral hemorrhage, hyphema, periocular swelling, globe rupture, and globe proptosis was also recorded. Lingual trauma and pharyngeal swelling were also noted.

### 2.5 Demographic data

Sex (male and female, intact or neutered) and age at the time of injury were recorded. Breed and skull shape were recorded. Weight at the time of injury and at the 2-week follow-up assessment was also recorded.

### 2.6 Treatment methods

Treatment methods were recorded based upon surgery reports. Methods recorded included rigid maxillomandibular fixation (RMMF), elastic maxillomandibular fixation (labial button technique, orthodontic buttons and chains, muzzle therapy) (EMMF), circummandibular cerclage wire, interdental wire and composite splint, interfragmentary wire, open reduction and internal fixation (ORIF) with 2.0 mm titanium locking miniplates (DePuy Synthes Vet, West Chester, PA), soft tissue closure alone, and salvage mandibulectomy (Table 2). For cats placed in MMF, restoration of pre-injury occlusion after apparatus placement was confirmed by ensuring that one-half to one-third of the height of the crowns of any present maxillary or mandibular canine teeth overlapped in a normal anatomic position. For cases in which an interdental wire and composite splint was applied, cats were extubated intra-operatively and then reintubated to ensure restoration of pre-injury occlusion without the hinderance of an endotracheal tube. Extra-oral intubation techniques such as transmylohyoid or pharangotomy intubation were used in cats that underwent ORIF so that temporary MMF could be applied prior to implant application. The placement of an esophageal feeding tube was noted. Finally, the need for extractions or crown height reduction with endodontic therapy at the time of fracture fixation surgery was recorded.

**Table 2:** Summary of treatments utilized for fracture fixation in the study group.

Method of fixation	Total number	Percentage (%)
Rigid MMF	25	22.9
Elastic MMF/Labial buttons/muzzle	19	17.4
Interdental wire and composite splint	12	11.0
Circummandibular cerclage wire	54	49.5
ORIF	13	11.9
Soft tissue closure	14	12.8
Salvage mandibulectomy, condylectomy	4	3.7
Supportive care	9	8.3
No specific treatment/Euthanasia	2	1.8

MMF = maxillomandibular fixation, ORIF = open reduction and internal fixation.

### 2.7 Healing evaluation

Recheck examination records were assessed for complications related to the trauma or to fixation. Clinical evaluation included assessment of presence of dehiscence, persistent malocclusion, pain, persistent anorexia or hyporexia, implant exposure, infection, reduced TMJ range of motion (or ankylosis), and failure of the apparatus used for fixation. Follow-up CT scans, when available, were evaluated by the same individuals evaluating the initial CT scans. Radiograph reports and radiographic images of follow-up examinations were also evaluated. Quality of fracture healing was given an ordinal score as performed in a previous study (21). Healing was deemed complete if no fracture was detected on follow-up CT (score = 0). Healing was deemed satisfactory if there was evidence of new bone formation across the fracture line and fracture fragments remained in alignment that did not hinder the patient’s function (score = 1). Healing was deemed unsatisfactory if there was no evidence of bone formation across the fracture line and/or the patient exhibited a significant malunion affecting masticatory function (score = 2). For symphyseal separation where osseous healing could not be evaluated due to the fibrocartilaginous nature of the mandibular symphysis, imaging findings were correlated with clinical examination findings for judgement of adequate healing. Follow-up records were reviewed for evidence of complications related to trauma or fixation.

### 2.8 Statistical methods

The Jonckheere-Terpstra trend test and Spearman correlation coefficients were used to evaluate the healing outcomes of fractures in relation to severity of fragmentation and displacement. Fisher’s exact test was used to evaluate the association of TMJ fractures with rostral mandibular trauma and to compare healing outcomes of various fixation techniques. Patient weight was tested for normality using the Shapiro–Wilk test and the Mann–Whitney U test was used to compare weight change in cats with esophageal feeding tubes versus cats without feeding tubes. Patients less than 5 months of age were excluded from weight comparison analyses. Time from initial 3D imaging scan to follow-up imaging scan for each treatment type was tested for normality using the Shapiro–Wilk test and the Mann–Whitney U test was used to compare time to follow-up imaging between the different treatment modalities. For all analyses, values of *p* < 0.05 were considered significant. All calculations were performed using Graph Pad Prism 10 Software (GraphPad, LaJolla, CA) and SPSS Statistical Software (IBM Version 30.0).

## 3 Results

### 3.1 Study population description

A total of 109 cats were included for evaluation. Sex distribution included 6 (5.5%) intact females, 15 (13.8%) intact males, 34 (31.2%) spayed females, and 54 (49.5%) castrated males. The mean age (SD) was 59.6 (55.1) months with an age range of 3- weeks-old to 16-years-old. Almost all cats were considered mesaticephalic with only 1 brachycephalic cat and no dolichocephalic cats included. The majority of cats were domestic short hair (*n* = 77, 70.6%), domestic medium hair (*n* = 10, 9.2%), or domestic long hair (*n* = 12, 11.0%). The majority (n = 63, 57.8%) of traumatic injuries were due to an unknown or unwitnessed cause. Known causes included animal altercation (*n* = 32, 29.3%), vehicular trauma (*n* = 9, 8.2%), fall from a height (*n* = 3, 2.7%), iatrogenic from surgical procedure (*n* = 1, 0.9%), and mandible becoming stuck in a collar (*n* = 1, 0.9%).

### 3.2 Commonly fractured locations

In total, 12 (11.0%) cats underwent initial imaging with conventional CT scan of the head while 97 (89.0%) underwent initial imaging with CBCT scan. Number of injured regions of the mandible ranged from 1 to 7 regions per cat with a mean (SD) of 3 (1.5) mandibular injuries per cat. A total of 100 (91.7%) cats had more than one region injured. Bilateral fractures were common, occurring in 42 (38.5%) cats. The most injured region of the mandible was the mandibular symphysis with 60 (55.0%) cats exhibiting symphyseal separation. The condylar process of the mandible and mid ramus region were the next most injured locations with 54 (49.5%) and 53 (48.6%) cats exhibiting fractures of these regions, respectively. In total, 46 (42.2%) cats exhibited condylar process fractures that involved the TMJ articular surface and 8 (7.3%) cats exhibited condylar process fractures that did not involve the articular surface (Figure 1).

### 3.3 Concomitant trauma

In total, 70 (64.2%) cats had concurrent maxillary trauma and 75 (68.8%) cats exhibited dentoalveolar trauma. Ocular trauma was noted in 40 (36.7%) cats and lingual trauma was noted in 23 (21.1%) cats. Evidence of traumatic brain injury was documented in 7 (6.4%) cats.

### 3.4 Treatment methods

In 54 (49.5%) cats, circummandibular cerclage wire was placed to treat symphyseal separation and fractures of the incisive region. RMMF was placed in 25 (22.9%) cats while EMMF was placed in 19 (17.4%) cats to treat fractures of the premolar, molar, and mid ramus regions. An interdental wire and composite splint were placed in 12 (11.0%) cats to treat fractures of the incisive, canine, and premolar regions. ORIF was performed in 12 (11.0%) cats to treat fractures of the premolar, molar, and mid ramus regions. A total of 4 (3.7%) cats were treated with salvage mandibulectomy or condylectomy while 14 (12.8%) cats were treated with soft tissue closure only. Nine (8.3%) cats were treated with supportive care only and had no surgical intervention (Table 2).

### 3.5 Complications

The most common complication following treatment was persistent malocclusion which occurred in 40 (36.7%) cats. Of those cats that developed a persistent malocclusion, 21 (52.5%) required treatment such as tooth extraction, crown height reduction with endodontic therapy, or odontoplasty to treat secondary soft tissue trauma related to the malocclusion. Of cats treated with interdental wire and composite splint, 50.0% (6/12) had a persistent malocclusion. Of cats treated with circummandibular cerclage wire alone, 22.7% (5/22) had a persistent malocclusion. Patients treated with RMMF and EMMF had a rate of persistent malocclusion of 54.5% (12/22) and 32.9% (9/17), respectively. One cat (0.9%) treated with ORIF exhibited persistent malocclusion. When comparing rate of persistent malocclusion in patients treated with MMF versus ORIF, patients treated with ORIF had a significantly lower prevalence of persistent malocclusion (53.9 and 9.1%, respectively, *p* = 0.0138). Eight (7.3%) cats in the entire study population were documented to have a broken splint or MMF device during the course of treatment. Six (5.5%) cats developed clinically significant malunion affecting occlusion and function. Maxillary lip entrapment occurred in 5 (4.6%) cats. Dehiscence and infection at the site of injury occurred in 3 (2.8%) cats and 2 (1.8%) cats, respectively. Two cats (1.8%) exhibited severe lingual injury from their initial trauma resulting in the inability to eat independently. Single cases of the following complications occurred: infection at the esophageal feeding tube site, subjectively decreased TMJ range of motion, odontodysplasia of a permanent successor tooth, and severe airway swelling requiring use of temporary tracheostomy. No cases of damage to tooth roots during implant placement were documented. No cases of TMJ ankylosis were recorded.

### 3.6 Overall survival

In total, 6 (5.5%) cats died or were euthanized during or around the time of assessment and treatment of maxillofacial trauma. Three (2.8%) cats were recommended to be euthanized, 1 cat due to the severity of concurrent traumatic brain injury, and 2 cats due to the severity of maxillofacial trauma and owner finances. One cat died due to a presumed aspiration episode after placement of RMMF. One cat developed concurrent ureteral obstruction and acute kidney injury. Finally, one cat suffered cardiorespiratory arrest of unknown cause during treatment. Mean (SD) follow up time for surviving cats was 69 (57.9) days.

### 3.7 Fracture healing

A total of 49 (45.0%) cats underwent follow-up CBCT scan while 2 (1.8%) cats underwent follow-up conventional CT scan. Dental radiographs were performed as a sole follow-up imaging modality in 22 (20.2%) cats. Mean (SD) time from initial imaging to follow up was 46.7 (22.8) days. When considering cats that underwent follow-up CBCT, there was a statistically significant, moderate positive correlation with fracture healing of the mandibular mid ramus and pre-operative displacement of fracture fragments of the mid ramus (T_JT_ = 249.5, *p* = 0.001, *ρ*_(s)_ = 0.564, *p* = <0.001). Similarly, there was a statistically significant, moderate positive correlation with fracture healing of the coronoid process and pre-operative displacement of fracture fragments of the coronoid process (T_JT_ = 86.0, *p* = 0.044, ρ_(s)_ = 0.451, *p* = 0.040) (Table 3). There were no statistically significant correlations detected for healing outcomes and pre-operative fragmentation for any regions of the mandible (Table 4).

**Table 3:** Data output for Spearman’s correlation for correlation between pre-operative displacement and quality of healing for each region of the mandible.

Displacement correlation with healing			
Region	Sample number	Spearman’s rho	*p* value (Spearman)
Incisive	20	0.257	0.274
Canine	7	0.242	0.602
Premolar	11	0.447	0.168
Molar	10	−0.126	0.729
Mid ramus	35	0.564	<0.001**
Coronoid process	21	0.451	0.040**
Condylar process	30	0.245	0.192

Statistically significant *p* values are marked with double star asterisk symbols. There was a statistically significant positive, moderate correlation between initial fracture displacement and healing quality for fractures of the mid ramus and coronoid process.

**Table 4:** Data output for Spearman’s correlation for correlation between pre-operative fragmentation and quality of healing for each region of the mandible.

Fragmentation correlation with healing			
Region	Sample number	Spearman’s rho	*p* value
Incisive	20	0.403	0.403
Canine	7	0.091	0.846
Premolar	11	0.311	0.351
Molar	10	0.327	0.356
Mid ramus	35	−0.118	0.501
Coronoid process	21	0.17	0.46
Condylar process	30	0.173	0.36

No statistically significant or clinically significant relationships between fragmentation and healing quality were noted.

### 3.8 Intervention and healing outcome

**Figure 4:**
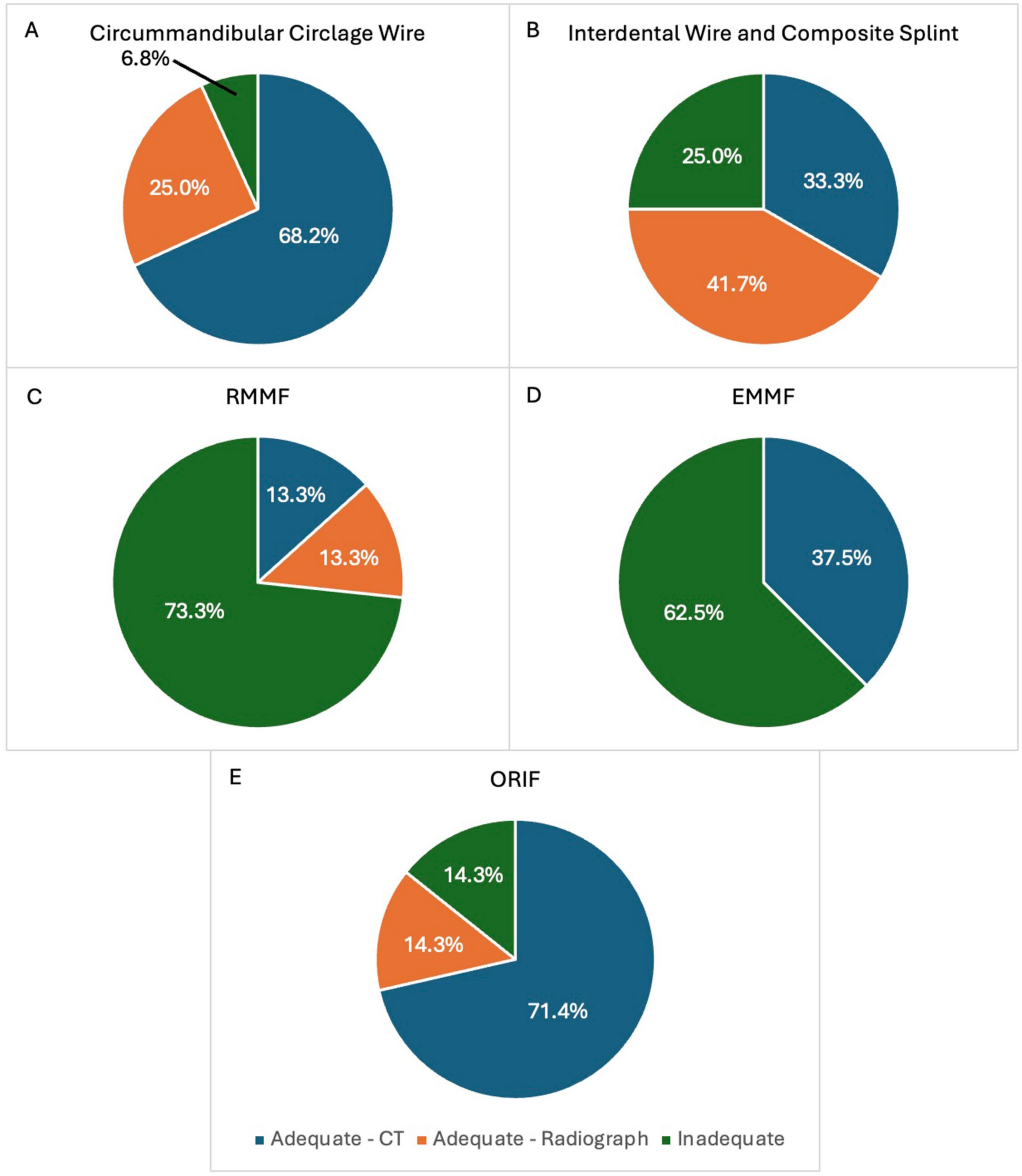
**(A–E)** Healing rates associated with each type of fracture fixation technique commonly used in the study population. The proportion of patients that exhibited adequate healing based on follow-up CT are shown in dark blue. The proportion of patients that exhibited adequate healing based upon follow-up radiographs are shown in orange. The proportion of patients that exhibited inadequate healing on follow-up CT or radiographs are shown in green. RMMF = rigid maxillomandibular fixation, EMMF = elastic maxillomandibular fixation, ORIF = open reduction and internal fixation.

**Figure 5:**
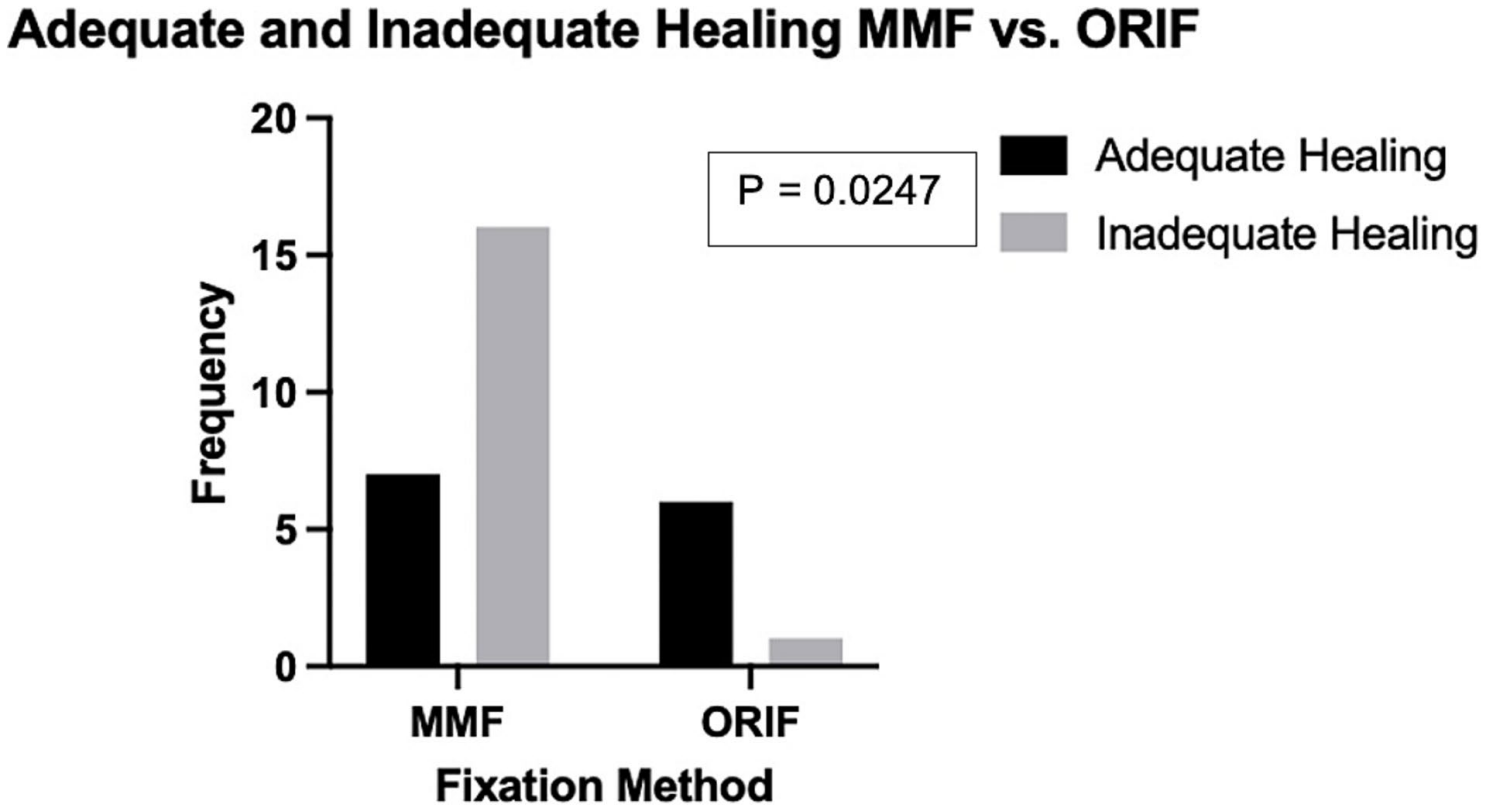
Comparison of the prevalence of adequate and inadequate healing based on follow-up imaging in patients treated with maxillomandibular fixation versus open reduction and internal fixation for fractures of the molar or mid ramus region of the mandible. Patients treated with ORIF displayed a higher rate of adequate healing as determined by follow-up diagnostic imaging.

**Figure 6:**
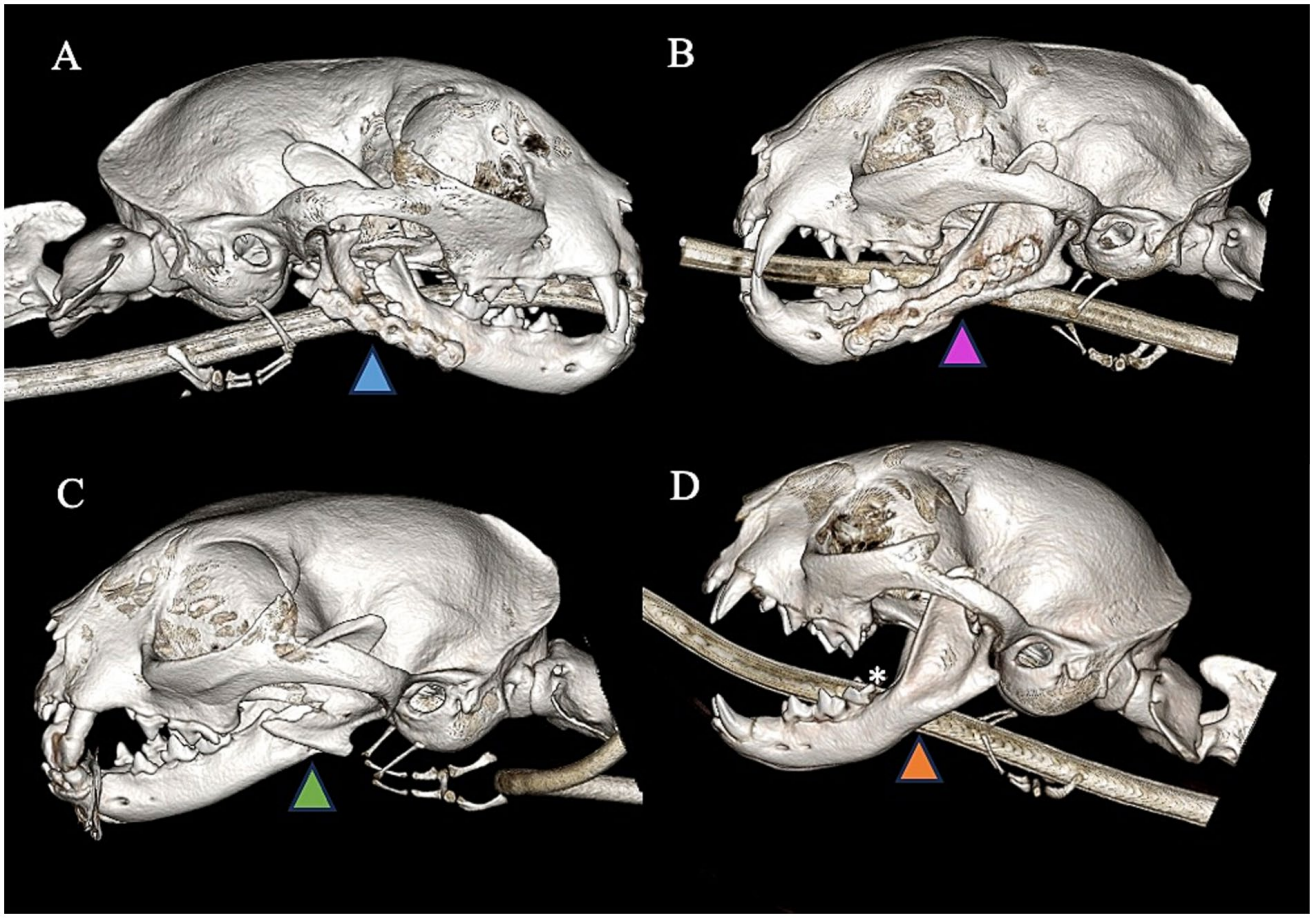
Right lateral view **(A)** of CBCT 3D reconstruction image demonstrating a cat treated with 2.0 titanium locking miniplate and exhibiting persistent fracture gap with no evidence of appropriate healing (blue arrowhead). The cat is in appropriate occlusion and function but lacks radiological evidence of bone healing. Left lateral view **(B)** of CBCT 3D reconstruction demonstrating a cat treated with 2.0 titanium locking miniplate and exhibiting excellent bone healing and remodeling of previous fracture (purple arrowhead). Left lateral view **(C)** of CBCT 3D reconstruction demonstrating a cat treated with RMMF and exhibiting improper healing and lack of bridging of fracture fragments (green arrowhead) as well as malocclusion. Left lateral view **(D)** of CBCT 3D reconstruction demonstrating a cat treated with EMMF and exhibiting excellent healing of previous fracture (orange arrowhead). There is also concurrent odontodysplasia of the distal aspect of the crown of the left mandibular first molar tooth (white asterisk).

Median time from initial imaging to follow-up imaging for each apparatus was determined to be the following: MMF 33.5 days, interdental wire and composite splint 48 days, circummandibular cerclage wire 43 days, and ORIF 48 days. Follow-up time was significantly shorter for cases that were treated with MMF when compared to cases that were treated with ORIF (*U* = 28.5, *n* = 30, *n* = 7, *p* = 0.0017). When considering patients that underwent diagnostic imaging follow-up, 41 (93.2%) patients treated with circummandibular cerclage wire exhibited appropriate healing (Figure 4A). In total, 9 (75%) patients treated with interdental wire and composite splint exhibited appropriate healing (Figure 4B). A total of 4 (26.7%) patients treated with RMMF and 3 (37.5%) patients treated with EMMF showed appropriate healing (Figures 4C,D, respectively). Altogether, 6 (85.7%) patients treated with ORIF showed appropriate healing on follow-up diagnostic imaging (Figure 4E). When pooling together different types of MMF and comparing to ORIF for the treatment of caudal mandibular fractures, ORIF had a significantly higher rate of adequate healing (30.4 and 85.7% respectively, *p* = 0.0247) (Figures 5, 6A–D).

### 3.9 Temporomandibular joint fractures

In total, 37 (33.9%) cats exhibited simultaneous injuries of the rostral mandible (symphyseal separation, incisive fractures, canine teeth region fractures) and condylar process. There was no statistically significant correlation between the occurrence of rostral mandibular injuries and condylar fractures (*p* = 0.5292).

### 3.10 Additional medical intervention

**Figure 7:**
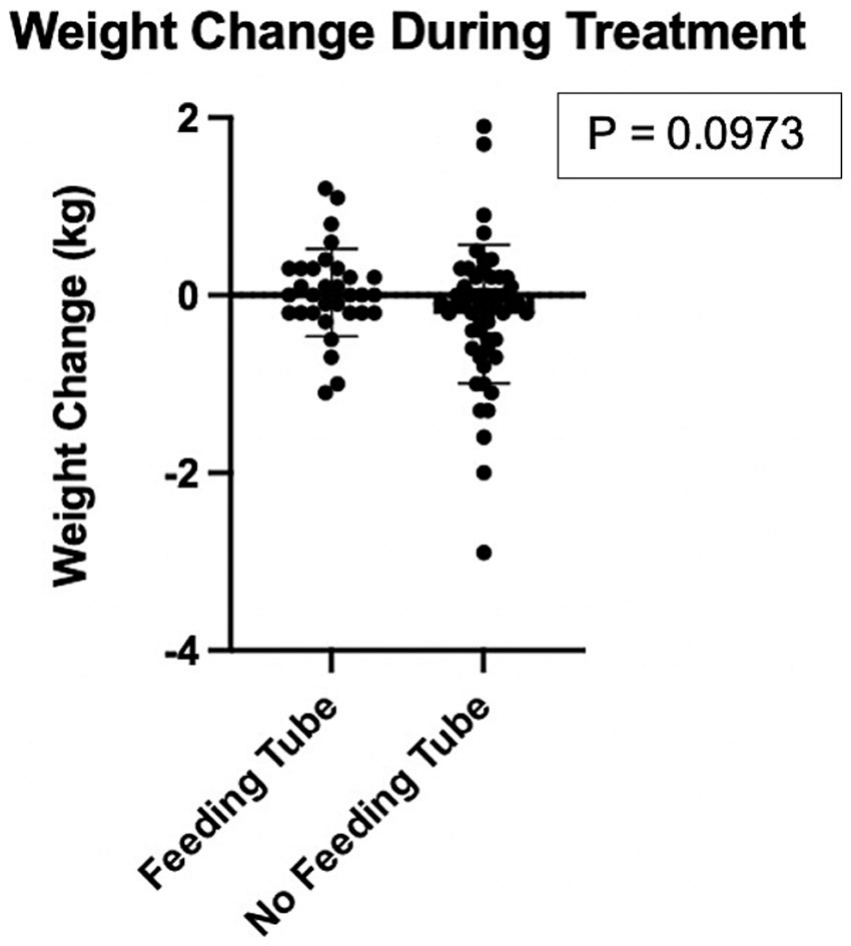
Comparison of weight change in kilograms of cats that had an esophageal feeding tube placed versus cats that did not have an esophageal feeding tube placed. Each black circle represents one cat. There was no statistically significant difference in weight change for the group of cats that received a feeding tube when compared to cats that did not receive a feeding tube.

A total of 61 (56.0%) cats had an esophageal feeding tube placed at the time of treatment. After excluding cats less than 5-months-old from analysis, no significant difference in weight gained or lost during the course of treatment was found between patients with a feeding tube when compared to patients without a feeding tube (*p* = 0.0973) (Figure 7).

## 4 Discussion

The present study represents the largest retrospective study to date focusing on treatment, clinical outcomes, and diagnostic imaging outcomes of mandibular trauma in cats. Our data demonstrated that the most commonly injured location was the mandibular symphysis followed by the condylar process and the mid ramus region. Second, these data showed that pre-operative increased fracture displacement in the mid ramus and coronoid process regions were correlated with unsatisfactory healing in those regions. Third, we demonstrated that patients treated with ORIF had better outcomes as evidenced by a significantly higher rate of adequate healing and lower prevalence of persistent malocclusion. Finally, the survival rate of cats was very high, suggesting that, depending on the fracture fixation method and peri-operative medical management, mandibular fractures in cats have a good to excellent prognosis.

The mandibular symphysis was the most commonly injured location of the mandible in the cat, likely due to the lower strength and stiffness of a fibrocartilaginous symphysis compared to that of cortical bone (22). The condylar process of the mandible and mid ramus region of the mandible were also injured in roughly half of the cats in the present cohort. Leveraging forces in the rostral mandible and the caudal position of the condylar process and the mid ramus resulting in longer moment arm likely contributed to the high rate of injury of the condylar process. Contrary to previous studies on maxillofacial trauma in dogs, rostral mandibular injuries were not statistically correlated with injuries in the caudal mandible in this cohort (21, 23). In agreement with previous studies, fractures involving the TMJ were a common occurrence in the cats included in the present study which underscores the necessity for advance diagnostic imaging to properly characterize maxillofacial trauma injuries (7, 10). Advanced diagnostic imaging, in the form of CT or CBCT techniques are known to be more sensitive for detecting traumatic injuries and important anatomic structures in the maxillofacial region of small animals as compared to skull radiographs (24, 25). A major consequence of undiagnosed or mischaracterized fractures involving or adjacent to the TMJ is intra-articular or extra-articular ankylosis which may require treatment involving ostectomy of large portions of bone or joint replacement surgery to preserve masticatory function (8, 9, 15, 26). None of the cats in the study group exhibited intra-articular or extra-articular ankylosis, likely due to the immediate attention to the fracture and the recommendation for physical therapy to maintain TMJ range of motion in cases of fracture of the condylar process. Fifteen cats with TMJ injuries were less than 1 year of age at the time of injury which is thought to be the population at highest risk of trauma related TMJ ankylosis (9).

We demonstrated that a higher degree of fracture fragment displacement is moderately correlated with worse fracture healing outcome in the mid ramus and coronoid process regions. These two regions differ from others in that they commonly experience marked displacement of fracture fragments and may not be treatable with direct fixation, depending on fracture location and configuration. When considering other regions of the mandible such as the rostral and tooth bearing regions, choice in fracture fixation technique had a greater impact on likelihood of successful healing than initial fracture configuration. This is because fixation techniques such as interdental wire and composite splint and circummandibular cerclage wire generally are successful in maintaining good fracture reduction and acceptable levels of strain when placed appropriately (17).

When considering fixation techniques, the location of the injured region, the anatomic constraints of placement of an apparatus, and fracture biomechanics play into a clinician’s choice in pursuing a given technique. For rostral mandibular fractures of the symphyseal and incisive region, circummandibular cerclage wire proved to be predictably successful. Similarly, interdental wire and composite splinting exhibited a high rate of healing based on follow-up diagnostic imaging, consistent with recently reported data in another study (17). The only fracture fixation techniques that could be directly compared were ORIF and MMF, as both were specifically utilized to treat fractures of the premolar, molar, and mid ramus region of the mandible. ORIF had a much higher success rate when compared to MMF, as ORIF not only provides proper fracture fragment reduction and stabilization but also a comparatively low strain environment. MMF is considerably less invasive and preserves the periosteum. However, MMF variably allows for reduction of fracture fragments and prohibits quick return to normal function. Hence, lack of physiologic bone stresses results in minimal or no mechanotransduction to stimulate bone regeneration and remodeling. Especially in cases of EMMF, strain at the fracture site is likely very high. However, it is likely that case selection may have influenced the comparative success rate as it is possible that fractures that would have been anatomically more challenging to treat with ORIF were by default placed in MMF. Additionally, median time to follow-up was significantly longer for cases treated with ORIF, allowing more time for healing. Despite the difference in follow-up times, some evidence of healing including callus formation, narrowing of fracture line, or disappearance of the fracture line is expected in dogs at 20–30 days following orthopedic fracture fixation (27). No specific fracture healing timeline is known for fractures of the mandible in cats. These comparative data suggest that there is a more predictable positive outcome with the use of ORIF for the treatment of caudal mandibular fractures in cats. Therefore, ORIF should be offered as a primary treatment over MMF whenever anatomically and technically feasible.

Overall survival rate was very high in the study population compared to previous studies examining maxillofacial trauma and feline trauma in general for a few reasons (5, 6, 11, 12). First, the present study focused on cats suffering from mandibular trauma and these patients may have less risk of immediate life-threatening injury such as traumatic brain injury compared to cats presenting for maxillofacial trauma in general. Furthermore, inclusion criteria confined study entrance to cats that received a conventional CT or cone beam CT scan. This parameter would inherently select for cats with caretakers that could invest in advanced veterinary care and were therefore more likely to pursue treatment. Additionally, institutional practices such as delaying OMFS trauma diagnostic testing and treatment for 24–48 h to allow for proper systemic evaluation and stabilization of the patient may have also contributed to the high survival rate (28, 29). It is also possible that the study population was biased toward patients that were able to survive to referral to a tertiary referral center and were stable enough to survive to discharge. Factors contributing to the demise of patients in the present group included concurrent injuries (brain injury, lingual trauma, other maxillofacial trauma) and other systemic complications. Roughly one-third of cats developed a persistent malocclusion following mandibular fracture and about one-half required some additional intervention to treat the consequences of the malocclusion. Specifically, when comparing MMF and ORIF, patients treated with ORIF had a much lower prevalence of persistent malocclusion. During proper placement of titanium implants, the patient is placed into temporary MMF during surgery to assist with alignment of fracture fragments and to ensure proper occlusion post-operatively (30). Thus, ORIF has a major benefit compared with MMF in that persistent malocclusion is a relatively rare finding post-operatively. Additionally, one patient that was treated with RMMF died shortly after treatment, likely related to an aspiration event precipitated by the choice in fracture repair. To conclude, these data suggest that mandibular trauma in cats has a good to excellent prognosis for good quality of life and return to function and that ORIF has superior outcomes for treatment of caudal mandibular fractures when compared with MMF.

The use of an esophageal feeding tube was a common recommendation in the study cohort but did not necessarily impact the ability for a given cat to maintain weight post-operatively. The decision to place a feeding tube in patients following maxillofacial trauma stems from the nature of the chosen fracture fixation technique and the cat’s ability to maintain nutrition and energy requirements given the constraints of the fixation apparatus. As such, feeding tubes are recommended in cases of MMF but are only occasionally needed in cases in which interdental wire and composite splints and ORIF are used. The use of a nasogastric tube is typically not recommended in maxillofacial trauma patients as there is a risk of insertion of the tube through unseen skull-base fractures into the intra-cranial space (31). To the authors’ knowledge, intra-cranial placement of a nasogastric tube in cases of maxillofacial trauma has not been reported in the veterinary literature but remains a possibility due to the proximity of the cribriform plate, olfactory and frontal lobe to the nasal cavity in the cat. Furthermore, patients may require nutrition supplementation for several days to weeks and discharge from the hospital with nasogastric tubes is not recommended due to risk of dislodgement and subsequent aspiration of food materials. Finally, medications can be more easily administered through larger diameter esophageal feeding tubes when compared with nasogastric tubes (32).

Limitations of the present study include non-standardized fracture configurations and post-operative care. Given the retrospective nature of the study, follow-up data was not standardized and surgical techniques likely varied from one clinician to another. In cats that were only included based on one non-anesthetized follow-up, medium-term and long-term complications may have not been noted. Also, a subset of cases was evaluated only with radiography and subtle indications of lack of healing may have been missed. Furthermore, some known fracture fixation techniques such as interfragmentary wire, absorbable plates, and different titanium plate configurations were not exhibited in the data set (16, 33). Additionally, individuals reviewing follow-up CT to determine outcome could not be blinded to treatment in most cases. Follow-up imaging may have also selected for cats that had post-operative complications, thereby inflating rates of unsatisfactory healing. Finally, the cats included in the study may have represented more severe injuries and as such, were referred to a tertiary care facility. Therefore, the study population may not reflect the standard degree of injuries in the emergency, urgent care, or other specialty setting.

In conclusion, the current retrospective study characterized the injured locations in the mandible of cats that sustained maxillofacial trauma and evaluated the success rates of minimally invasive and invasive treatments. We demonstrated a higher success rate when utilizing ORIF when compared to MMF for the treatment of caudal mandibular fractures as exhibited with quick return to normal function, lack of malocclusion and bone healing. We also found an overall good success rate for interdental wire and composite splint and circummandibular cerclage wire. Finally, cats in this study exhibited a high survival rate, suggesting that while mandibular fractures can be acutely debilitating, there is a good prognosis when appropriate diagnostic work-up and treatment are pursued.

## Data Availability

The raw data supporting the conclusions of this article will be made available by the authors, without undue reservation.
